# Orosensory Detection of Dietary Fatty Acids Is Altered in CB_1_R^−/−^ Mice

**DOI:** 10.3390/nu10101347

**Published:** 2018-09-21

**Authors:** Léa Brissard, Julia Leemput, Aziz Hichami, Patricia Passilly-Degrace, Guillaume Maquart, Laurent Demizieux, Pascal Degrace, Naim Akhtar Khan

**Affiliations:** 1 Physiology of Nutrition and Toxicology (NUTox), INSERM UMR U1231, Université de Bourgogne Franche-Comté/Agro-Sup, 21000 Dijon, France; Lea.Brissard@u-bourgogne.fr (L.B.); Julia.Leemput@u-bourgogne.fr (J.L.); Aziz.Hichami@u-bourgogne.fr (A.H.); Patricia.Degrace@u-bourgogne.fr (P.P.-D.); Guillaume.Maquart@u-bourgogne.fr (G.M.); 2 Pathophysiology of Dyslipidemia (PADYS), INSERM UMR U1231, Université de Bourgogne Franche-Comté/AgroSup, 21000 Dijon, France; Laurent.Demizieux@u-bourgogne.fr (L.D.); Pascal.Degrace@u-bourgogne.fr (P.D.)

**Keywords:** nutrition, lipids, fat taste, CD36, feeding behavior, cannabinoids, CB_1_R, GLP-1

## Abstract

Obesity is one of the major public health issues, and its prevalence is steadily increasing all the world over. The endocannabinoid system (ECS) has been shown to be involved in the intake of palatable food via activation of cannabinoid 1 receptor (CB_1_R). However, the involvement of lingual CB_1_R in the orosensory perception of dietary fatty acids has never been investigated. In the present study, behavioral tests on CB_1_R^−/−^ and wild type (WT) mice showed that the invalidation of *Cb_1_r* gene was associated with low preference for solutions containing rapeseed oil or a long-chain fatty acid (LCFA), such as linoleic acid (LA). Administration of rimonabant, a CB_1_R inverse agonist, in mice also brought about a low preference for dietary fat. No difference in CD36 and GPR120 protein expressions were observed in taste bud cells (TBC) from WT and CB_1_R^−/−^ mice. However, LCFA induced a higher increase in [Ca^2+^]_i_ in TBC from WT mice than that in TBC from CB_1_R^−/−^ mice. TBC from CB_1_R^−/−^ mice also exhibited decreased *Proglucagon* and *Glp-1r* mRNA and a low GLP-1 basal level. We report that CB_1_R is involved in fat taste perception via calcium signaling and GLP-1 secretion.

## 1. Introduction

Due to the abundance of food resources in the modern era, the Western diet is comprised of more than 40% of fat, thereby contributing to the increase in the prevalence of obesity that is associated with a number of pathologies (type 2 diabetes mellitus, hypertension, cancer, and others).

The taste modalities represent an essential factor involved in food intake. It is now well established that obese subjects exhibit higher spontaneous preference for fat than lean subjects [1,2]. Recent studies have proposed the existence of a sixth taste modality dedicated to the orosensory perception of dietary fat. The CD36 (cluster of differentiation 36) has been suggested to act as lingual lipid receptor [3]. The binding of a fatty acid to lingual CD36 in taste bud cells (TBC) leads to modifications in the membrane potential and to an increase in free intracellular calcium concentrations, [Ca^2+^]_i_, followed by the release of neurotransmitters [4,5]. These gustatory signals are transmitted from the oral cavity, through the cranial nerve IX (lingual branch of the glossopharyngeal), to the nucleus of the solitary tract (NST) [6]. The NST is connected to different brain areas associated with food intake, rewarding, memory, and processes integrating visceral signals [7,8]. Hence, the integration of the gustatory signals in brain triggers a behavioral and metabolic response [8].

GPR120 (G protein-coupled receptor 120) has also been proposed to play a role in fat-related regulation of satiation [8,9]. Nevertheless, GPR120 does not seem to have a major role in oral fat detection. Indeed, contradictory results have been reported for behavioral tests in GPR120^−/−^ mice [10,11,12]. However, the implication of GPR120 in the release of the incretin hormone glucagon-like peptide-1 (GLP-1) has been highlighted in mouse taste bud cells [9,13]. Thus, CD36 is likely to play a role in the detection of dietary fatty acids in TBC, whereas GPR120 would be implicated in the modulation of postprandial fat taste sensitivity.

The presence of GLP-1 and its receptor in the gustatory mucosa has been demonstrated [9,14,15], suggesting that taste bud cells may modulate taste perception in an autocrine or a paracrine manner. Indeed, linoleic acid (LA) has been reported to induce GLP-1 release in human TBC in a GPR120-dependent manner [9]. Martin et al. [13] have suggested that GLP-1 is locally active and might affect the basic functions in mouse taste buds. Besides, Shin et al. [15] showed that local GLP-1 signaling could enhance sweet-taste sensitivity, supporting the existence of a paracrine mechanism for the regulation of taste function.

The implication of the endocannabinoid system in the regulation of food intake is well documented [16,17,18,19,20]. Several studies have demonstrated that exogenous cannabinoids, like delta 9-tetrahydrocannabinol (Δ9-THC) or anandamide (AEA), induce hyperphagia and preference for palatable food [18,19] via cannabinoid-1 receptors (CB_1_R) [21]. Therefore, the CB_1_R blocker/inverse agonist, rimonabant, has been used in the treatment of obesity [22,23,24].

Being largely expressed in the central and peripheral nervous system, CB_1_R has also been detected in TBC, and it has been shown that the activation of these receptors by cannabinoids enhances sweet taste [7]. However, the involvement of lingual CB_1_R in fat taste perception has never been investigated. Considering that the increase in dietary fat intake plays an important role in the prevalence of obesity, the present investigation was designed to assess whether the activation of CB_1_R in TBC is associated with the altered orosensory perception of dietary lipids in CB_1_R^−/−^ and wild type (WT) mice.

## 2. Materials and Methods 

### 2.1. Ethical Approval

French guidelines for the use and care of laboratory animals were followed, and the experimental protocols were approved by the regional animal ethic committee of the University of Burgundy. In vivo studies were performed on male C57BL/6J wild type (WT) mice (Janvier Labs, Le Genest Saint Isle, France) and CB_1_R^–/–^ mice (generous gift from Dr. James Pickel, National Institute of Mental Health, Bethesda, MD, USA) with a C57BL/6J background. Animals were individually housed in a controlled environment (constant temperature and humidity, dark period from 19:00 to 7:00). The mice had free access to standard regular chow and tap water during the experiments, unless otherwise specified. 

### 2.2. Behavioral Experiments 

#### 2.2.1. Two-Bottle Preference Tests

After being deprived of water for 6 h, mice were offered simultaneously two bottles, containing either control or experimental solution for 12 h. To minimize bias due to textural properties, the two solutions contained 0.3% xanthan gum (*w*/*v*, Sigma, Saint Quentin-Fallavier, France), whereas the experimental solutions were added with either 0.2% rapeseed oil (*w*/*v*, Fleur de Colza, Lesieur, France) or 0.2% linoleic acid (*w*/*v*, LA, Sigma). At the end of each test, the intake of control and experimental solutions was recorded by weighing the feeders/bottles. The experiments were repeated two times, independently.

In parallel, two groups (*n* = 5 each) of 10 WT mice, treated daily with rimonabant (SR141716, 10 mg/kg of body weight, Sanofi, Paris, France) or vehicle (0.1% DMSO/0.025% Tween 80 in 0.9% NaCl), by an intraperitoneal injection for 26 days, were subjected to the same two-bottle preference test. Food intake and weight were monitored during the experiment.

#### 2.2.2. Licking Tests

The CB_1_R^−/−^ (*n* = 9) and WT (*n* = 9) mice were deprived of food and water for 6 h before the test. The mice were conditioned to choose between a palatable (4% sucrose) and a control solution. Once the mice were conditioned, they were randomly subjected to two-bottle test, containing either control (0.3% xanthan gum) or a test solution (0.3% xanthan gum + 0.2% linoleic acid, LA). The number of licks, motivated by each bottle, were recorded using computer-controlled lickometers (Med Associates, Fairfax, VT, USA). Data were analyzed for 5 min from the first lick. 

### 2.3. Papillae and Taste Buds Isolation 

The mice were anesthetized with 2% isoflurane gas, and then sacrificed by cervical dislocation. Taste bud cells (TBC) were isolated according to previously published procedure [4]. In brief, lingual epithelium was separated from connective tissues by enzymatic dissociation (elastase and dispase mixture, 2 mg/mL each in Tyrode buffer: 120 mM NaCl, 5 mM KCl, 10 mM HEPES, 1 mM CaCl_2_, 10 mM glucose, 1 mM MgCl_2_, 10 mM Na pyruvate, pH 7.4). Samples were frozen immediately in liquid nitrogen and stored at −80 °C (not exceeding one month) until RNA extraction, or lysed in a buffer for Western blot analyses. For real-time qPCR and Western blot, each point corresponds to a pool of TBC from four mice.

### 2.4. Real-Time qPCR

Total RNA from CB_1_R^−/−^ and WT TBC (*n* = 6) was extracted by using TRIzol method according to the manufacturer’s recommendations (Invitrogen, Cergy-Pontoise, France). After purification, mRNA was resuspended in RNase free water. The samples were then analyzed and quantified using Traycell (Hellma Analytics, Müllheim, Germany). Samples having a purity (A_260_/A_280_) between 1.80 and 2.00 were retained for the rest of the experiment. mRNA (500 ng) was reverse-transcribed into cDNA using M-MLV reverse transcriptase (Invitrogen) in a 20 µL of reaction volume containing 5 µL mRNA, 1 µL random primer (100 ng/µL) (Invitrogen), 0.4 µL dNTP (25 mM), 8.6 µL RNase-free water. After incubation for 5 min at 65 °C, 2 µL 5× First-Strand Buffer, 1 µL DTT, 1 µL M-MLV (200 UI) and 1 µL RNaseOUT were added, and the samples were incubated for 1 h at 42 °C and then for 15 min at 70 °C. Real time qPCR reactions were performed on 10 ng cDNA in a 20 µL of reaction volume in triplicates with a StepOnePlus (Life Technologies, Saint-Aubin, France) device with the use of SYBR green PCR Master Mix (Life Technologies, Saint-Aubin, France). For each gene, a standard curve was established from five cDNA dilutions (50 ng to 0.05 ng per well) and used to determine the PCR efficiency. Forward and reverse primer sequences used for amplification were 5′-GGACACATGAAGTCATCTTTGCCT-3′ and 5′-CAAGCCCTGGAAGGAAGTGAAGGA-3′ for *Glp-1r* (NM_021332), 5′-TGCTGAAGGGACCTTTACCAGTGA-3′ and 5′-GCCTTTCACCAGCCAAGCAATGAA-3′ for *Gcg* (NM_008100), and 5′-TTCTTTGCAGCTCCTTCGTT-3′ and 5′-ATGGAGGGGAATACAGCCC-3′ for *β-actin* (NM_007393). The amplicon size for *Glp-1r* is 107 bp and is located in the exon 11 and 12; for *Gcg* is 85 bp and is located in the exon 4; for *β-actin* is 149 bp and is located in the exon 1 and 2. Real time qPCR reactions were performed with a denaturing step of 95 °C for 10 min, followed by 40 cycles of 95 °C for 15 s and 60 °C for 1 min. The primer specificity was checked using the melt curves. The PCR efficiency was calculated as follow 10^−1/slope^ − 1. The parameters for *GLP-1r* were as follows: slope −3.241, y intercept 33.848, *R*² 0.98, and PCR efficiency 1.03; *Gcg*: slope −3.527, y intercept 29.296, *R*² 0.92, and PCR efficiency 0.92; and *β-actin*: slope −2.837, y intercept 29.296, *R*² 0.996, and PCR efficiency 1.25. The comparative 2^−ΔΔCT^ method was used for relative quantification. 

### 2.5. Western Blotting

Freshly isolated mouse TBC were lysed using a micro-potter in 20 µL of TSE buffer (50 mM Tris HCl, 150 mM NaCl, 1 mM EDTA, 1% Nonidet P40, 5 µL/mL protease inhibitors (Sigma)) [25]. Samples were stored on ice for 30 min, and then centrifuged (10,000 *g*, 10 min, 4°C). Lysates were used immediately or stored at −80 °C until the assay. Protein concentrations in homogenates were assayed using the BCA assay (Sigma, Saint Quentin-Fallavier, France). Denatured proteins (25 µg) were separated by SDS-PAGE (8%) and transferred to a polyvinylidene difluoride membrane. After being blocked for 3 h using a TBS buffer containing 5% BSA and 0.05% Tween-20, the membrane was incubated overnight with either of the antibodies: anti-CD36 antibody (R&D Systems, AF2519; 1:1000), anti-GPR120 antibody (Abcam, Paris, France, ab97272; 1:500), anti-α-gustducin antibody (Santa Cruz, Heidelberg, Germany, sc-395; 1:200) and anti-β-actin antibody (Santa Cruz, Heidelberg, Germany, sc-47778; 1:5000). The α-gustducin was used as an internal reference protein. After a set of washes, the appropriate peroxidase-conjugated secondary antibody was added. Antibody labeling was detected by chemiluminescence (Clarity, Bio-Rad, Marnes-la-Coquette, France). 

### 2.6. Tissue Culture of TBC and GLP-1 Release

Papillae from WT and CB_1_R^−/−^ mice were isolated and incubated at 36 °C. The incubation media contained either 33 μM fatty acid-free BSA alone (control group) or 200 μM linoleic acid (LA) mixed and vortexed with 33 μM fatty acid-free BSA. After 2 h of incubation, the media were collected, and the active GLP-1 release was measured by ELISA (Millipore S.A.S., Molsheim, France). As the secretion of GLP-1 by TBC is very low, to be sure to detect active GLP-1 in the incubation medium, 10 pM of pure GLP-1 was systematically added in each experimental well, but not in standard curve, according to the manufacturers’ recommendations. The dipeptidyl peptidase 4 (DPP4) inhibitor (0.1%, Millipore) was added to the medium to prevent GLP-1 degradation.

### 2.7. Measurement of Ca^2+^ Signaling

TBC were freshly isolated from mouse tongues as described by Dramane et al. [26]. The cells were cultured onto 24-well plates, containing RPMI-1640 medium, supplemented with 10% fetal calf serum, 2 mM glutamine, 50 µg/mL penicillin–streptomycin, and 20 mM HEPES, and incubated overnight at 37 °C. The next day, the supernatant was discarded. The cells were then incubated with Fura-2/AM (Invitrogen) at 1 μM for 30 min at 37 °C in loading buffer which contained the following: 110 mM NaCl, 5.4 mM KCl, 25 mM NaHCO_3_, 0.8 mM MgCl_2_, 0.4 mM KH_2_PO_4_, 20 mM Hepes, 1.2 mM CaCl_2_, 10 mM Glucose; pH 7.4. After adding the test molecules into the wells, the changes in intracellular free Ca^2+^ (*F*_340_/*F*_380_) were monitored under the Nikon microscope (TiU) by using S-fluor 40× oil immersion objective. NIS-Elements software was used to record the images. The microscope was equipped with Lucas EM-CCD (Andor Technology, Gometz-le-châtel, France) camera for real-time recording of 16-bit digital images. The dual excitation fluorescence imaging system was used to analyze individual cells. The changes in intracellular free Ca^2+^ were expressed as ΔRatio, calculated as the difference between *F*_340_ and *F*_380_. All test molecules were added in small volumes with no interruption in recordings. For Ca^2+^ signaling experiments, the fatty acid was dissolved in ethanol (0.1%, *v*/*v*) and added into the experimental cuvette. 

Anandamide (AEA, CB_1_R endogenous ligand), arachidonyl-2′-chloroethylamide (ACEA, CB_1_R synthetic ligand), LA, DB-cAMP, and U73122 were supplied by Sigma (Saint Quentin-Fallavier, France). A784168, TRPV1 (transient receptor potential vanilloid 1) antagonist, and rimonabant (CB_1_R inverse agonist) were provided by Tocris (Bio-Techne, Lille, France) and Sanofi (Paris, France), respectively.

### 2.8. Statistics

Results are expressed as means ± SEM. The significance of differences between groups was evaluated with GraphPad Prism (GraphPad Software, La Jolla, CA, USA) using two-tailed Student’s *t*-test or two-way ANOVA with Bonferroni correction. A *p* value of less or equal 0.05 was considered to be statistically significant.

## 3. Results

### 3.1. The Absence of CB_1_R Gene Induces a Low Preference for Fatty Solutions Independently of Postprandial Factors

**Figure 1:**
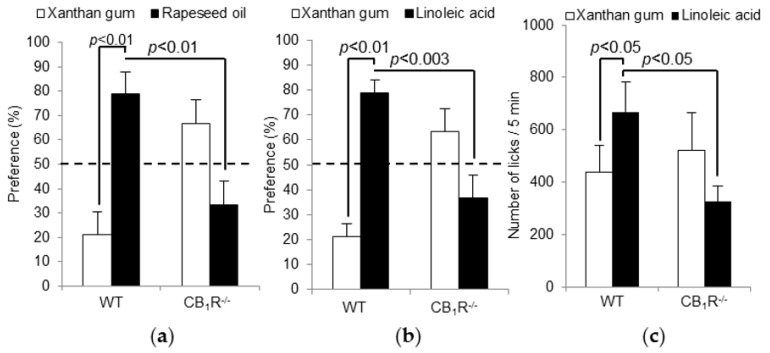
Effect of CB_1_R gene invalidation on preference for lipids. Two bottles (control and experimental) were simultaneously offered to wild type (WT) and CB_1_R^−/−^ mice for 12 h. The experimental solution contained (**a**) 0.2% of rapeseed oil (*w*/*v*); (**b**) 0.2% of linoleic acid (*w*/*v*) diluted in xanthan gum. The control solution contained 0.3% of xanthan gum (*w*/*v*). Values are expressed as mean ± SEM (*n* = 10). Dotted line represents the absence of preference in CB_1_R^−/−^ mice (less than 50% of preference). (**c**) Short-term (5 min) licking tests in WT and CB_1_R^−/−^ mice were performed as described in Materials and Methods. Animals were subjected to a control solution (xanthan gum) and an experimental solution containing 0.2% of linoleic acid. Values are expressed as mean ± SEM (*n* = 9).

CB_1_R^−/−^ mice displayed a significant decrease in the preference for the fatty solutions (rapeseed oil and LA) compared to wild type mice (Figure 1a,b). Hormonal post-ingestive regulatory feedback greatly influences metabolism and appetite and, consequently, behavior during long-term two-bottle preference tests [27] under CB_1_R activation [28]. Therefore, we performed short-term licking tests to measure fat preference limiting post-ingestive cues. LA was chosen among other LCFAs because it showed the best results in two-bottle preference tests. As shown in Figure 1c, the number of licks for LA was significantly higher in WT mice than in CB_1_R^−/−^ mice, confirming the low preference in CB_1_R^−/−^ mice for fatty solutions.

### 3.2. Treatment with Rimonabant Induces a Low Preference for Fat Solutions and Does Not Alter Feeding Behavior

**Figure 2:**
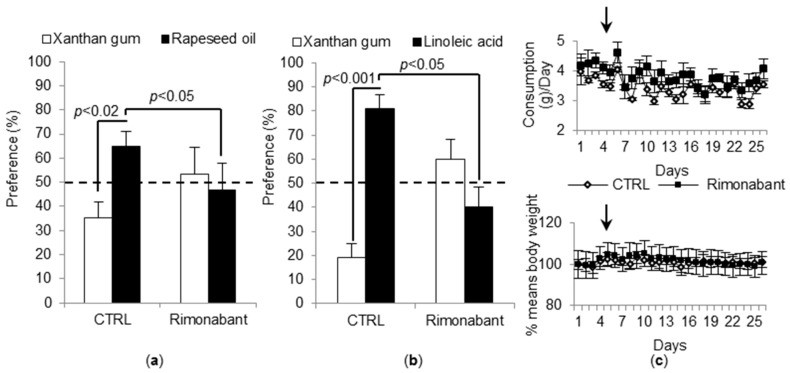
Effect of rimonabant on preference for lipids, body weight, and feeding behavior. (**a**,**b**) WT mice, treated with either rimonabant (10 mg∙kg^−1^∙day^−1^) or vehicle (CTRL), were simultaneously offered two bottles, a control one and an experimental one. The latter bottle contained either 0.2% of rapeseed oil (*w*/*v*) (**a**) or 0.2% of linoleic acid (*w*/*v*) (**b**) diluted in xanthan gum. Control solution contained 0.3% of xanthan gum. Values are expressed as mean ± SEM (*n* = 5). Dotted line represents the absence of preference in rimonabant-treated mice (less than 50% of preference). (**c**) Food intake and body weight variations in mice treated or not with rimonabant and fed a standard chow. Values are expressed as mean ± SEM (*n* = 5). Black arrows indicate the beginning of the treatment with rimonabant.

The mice treated with rimonabant for 26 days exhibited the same behavior as CB_1_R^−/−^ mice, i.e., a low preference for fatty solutions (Figure 2a,b). Interestingly, the mice treated with rimonabant did not show reduced food intake or body weight (Figure 2c). As the rimonabant induces an early and transient effect, we monitored these parameters for 5 days before starting two-bottle preference tests. Thus, no bias interfered with the behavioral experiments.

### 3.3. CD36 and GPR120 Protein Expressions Are Not Altered in TBC of CB_1_R^−/−^ Mice

**Figure 3:**
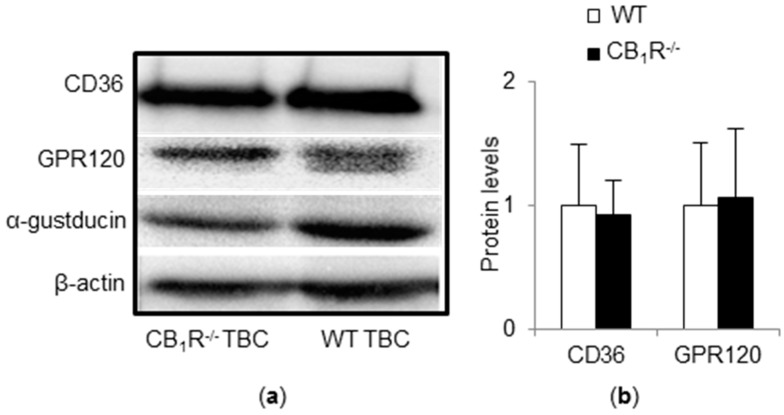
Impact of CB_1_R gene invalidation on CD36 and GPR120 protein expressions. CD36 and GPR120 protein levels were measured by Western blotting in taste bud cells (TBC) (*n* = 2) from WT and CB_1_R^−/−^ mice. (**a**) A representative blot corresponding to a pool of total proteins from four mice TBC is shown. (**b**) The corresponding histogram shows CD36 and GPR120 protein levels. Values are expressed as mean ± SD (*n* = 2).

CB_1_R gene invalidation did not interfere with CD36 and GPR120 protein expression in taste buds from CB_1_R^−/−^ (Figure 3). α-Gustducin, a marker of type II TBC, remained stable (Figure 3). It seems that the low preference for fatty solutions observed in CB_1_R^−/−^ mice was not due to altered expression of CD36 and GPR120. 

### 3.4. CB_1_R Gene Invalidation Induces a Decrease in Proglucagon and GLP-1r mRNA and Basal GLP-1 Level 

**Figure 4:**
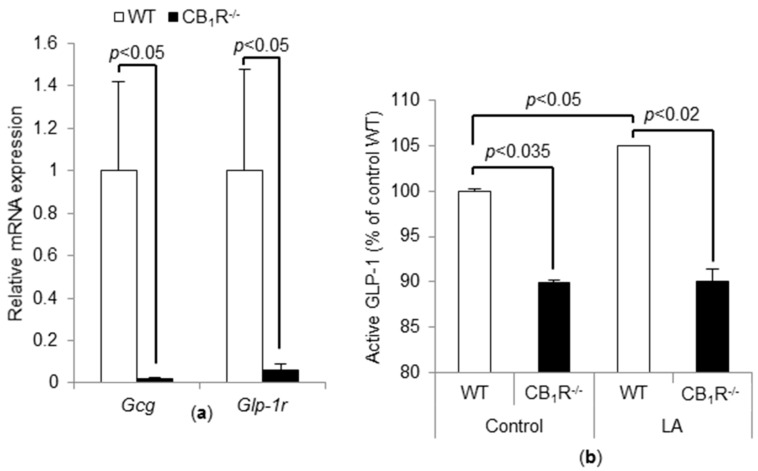
Effect of CB_1_R gene invalidation on GLP-1. (**a**) *Proglucagon* *(Gcg)* and *GLP-1r* mRNA levels were assayed by real-time qPCR in mouse TBC from WT and CB_1_R^−/−^ mice. Values are expressed as mean ± SEM (*n* = 6). (**b**) ELISA results showing GLP-1 release by freshly isolated mouse TBC incubated for 2 h in the presence of 33 µM fatty acid-free BSA alone (CTRL) or with 200 µM linoleic acid (LA). Each value corresponds to the GLP-1 released by cultured TBC. We independently reproduced the results twice, by using, each time, TBC from three mice. We observed identical results, and we pooled them. Each point represents values as mean ± SD (*n* = 6).

*Proglucagon* and *GLP-1r* mRNA levels were significantly lower in CB_1_R^−/−^ TBC than WT TBC (Figure 4a). According to previously published data [9], LA induces the release of GLP-1 from mouse TBC. To measure the release of active GLP-1, the mouse TBC were incubated for 2 h in an oxygenized medium containing anti-DPP4, to prevent GLP-1 degradation, and exposed, or not, to 200 µM LA. In CB_1_R^−/−^ mice TBC, GLP-1 release in the culture medium was significantly lower than that in WT TBC, in both basal and LA-stimulated conditions. As expected, LA induced a small, but significant, release of active GLP-1 in culture medium of WT TBC (Figure 4b). 

### 3.5. Both LA and Cannabinoids Induce CB_1_R-Dependent Ca^2+^ Responses in TBC

**Figure 5:**
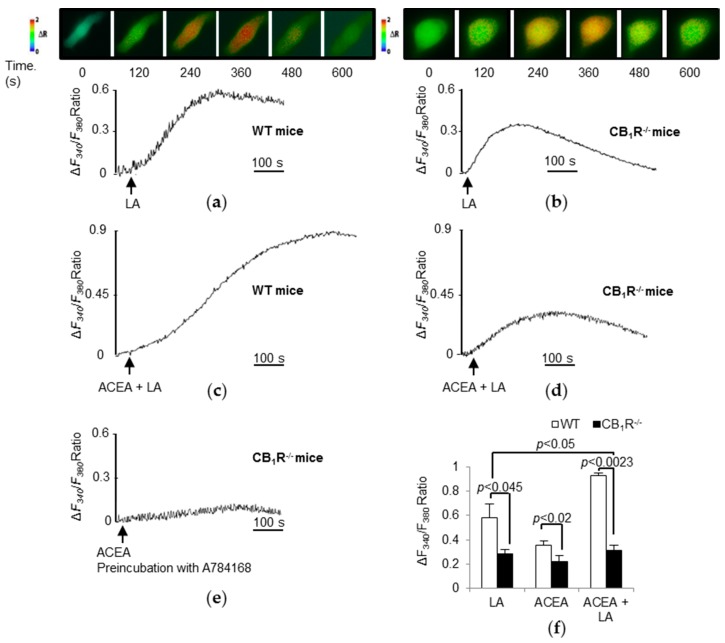
Effects of linoleic acid (LA) and cannabinoids on Ca^2+^ signaling in mouse TBC. Ca^2+^ imaging studies were performed in calcium-containing (100% Ca^2+^) buffer. The changes in free intracellular Ca^2+^ concentrations (Δ*F_340_*/*F_380_*) were monitored under the Nikon microscope (TiU) by using S-fluor 40× oil immersion objectives. Colored time-lapse changes show the kinetics of the rise in [Ca^2+^]_i_ levels in taste bud cells freshly isolated from WT mice (**a**) and CB_1_R^−/−^ mice (**b**) following addition of LA (25 µM) and the corresponding graphs below. Changes in [Ca^2+^]_i_ evoked by combined addition of ACEA (1.5 µM) and LA (25 µM) in WT (**c**) and CB_1_R^−/−^ TBC (**d**), respectively. Changes in [Ca^2+^]_i_ evoked by ACEA (1.5 µM) after a 15 min preincubation with A784168, a TRPV1 antagonist, in CB_1_R^−/−^ TBC (**e**). The arrowheads indicate when the test molecules were added. Variations in Δ*F*_340_/*F*_380_ Ratio induced by LA (25 µM), ACEA (1.5 µM), and ACEA (1.5 µM), in combination with LA (25 µM), in WT and CB_1_R^−/−^ mice TBC (**f**). Values are expressed as mean ± SEM (*n* = 5).

In mouse TBC, LCFA evokes increases in Ca^2+^ signaling [26]. As expected, LA triggered a higher rise in [Ca^2+^]_i_ in WT TBC [6] (Figure 5a,f) than that in CB_1_R^−/−^ TBC (Figure 5b,f). Similarly, arachidonyl-2′-chloroethylamide (ACEA, a specific CB_1_R agonist), induced a rise in [Ca^2+^]_i_ in WT TBC (Figure 5f). However, ACEA triggered a significantly lower increase in [Ca^2+^]_i_ in CB_1_R^−/−^ TBC than that in WT TBC (Figure 5f). Finally, the combination of LA and ACEA also induced a strong rise in [Ca^2+^]_i_ in WT TBC (Figure 5c,f) corresponding to the combined effect of the two molecules that was not apparent in CB_1_R^−/−^ TBC (Figure 5d,f). Interestingly, when tested on CB_1_R^−/−^ TBC, ACEA still triggered a rise in [Ca^2+^]_i_ (Figure 5f). Furthermore, blockade of TRPV1 with a specific antagonist, A784168, in CB_1_R^−/−^ TBC curtailed the action of ACEA on calcium response (Figure 5e).

### 3.6. CB_1_R Blockade Significantly Decreases Ca^2+^ Responses Triggered by LA, AEA, and ACEA in WT TBC 

**Figure 6:**
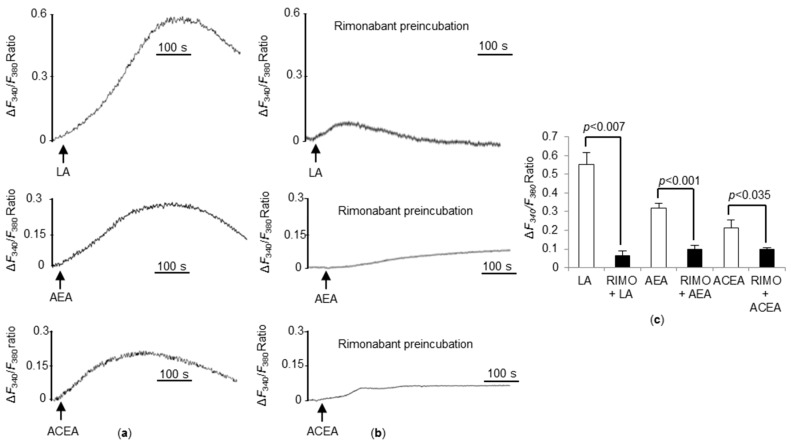
Effects of rimonabant on linoleic acid (LA) and cannabinoid-induced Ca^2+^ signaling in TBC. Ca^2+^ imaging studies were performed in calcium-containing buffer. The changes in free intracellular Ca^2+^ concentrations (Δ*F_340_*/*F_380_*) were monitored under the Nikon microscope (TiU) by using S-fluor 40× oil immersion objectives. Graphs show the increase in [Ca^2+^]_i_ in taste bud cells freshly isolated from WT mice following addition of LA (25 µM), anandamide (AEA, 5 µM), and ACEA (1.5 µM) (**a**). WT TBC before the addition of LA (25 µM), AEA (5 µM) and ACEA (1.5 µM) were preincubated (15 min) with rimonabant (50 µM) (**b**). The arrowheads indicate when the test molecules were added. Changes in Δ*F_340_*/*F_380_* Ratio induced by LA (25 µM), AEA (5 µM), and ACEA (1.5 µM) in WT mice TBC after a preincubation with or without rimonabant (RIMO, 50 µM) (**c**). Values are expressed as mean ± SEM (*n* = 5).

To further explore the role of CB_1_R on calcium signaling, we preincubated WT TBC with a specific CB_1_R inverse agonist rimonabant. As observed in Figure 6, rimonabant significantly abrogated [Ca^2+^]_i_ responses, induced by LA, AEA, and ACEA (Figure 6c), corroborating the previous results observed in CB_1_R^−/−^ TBC. 

### 3.7. AEA-Induced Ca^2+^-Signaling Is PLC Dependent 

**Figure 7:**
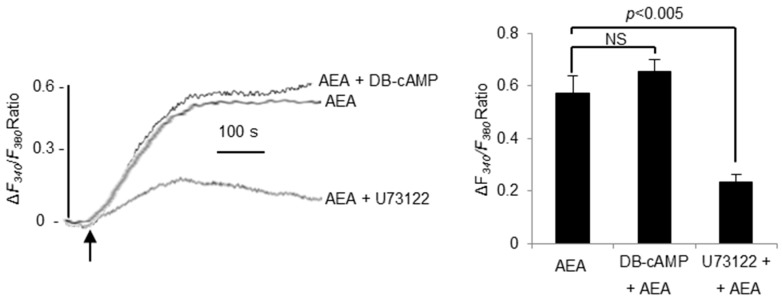
Effects of DB-cAMP and U73122 on anandamide (AEA, 5 µM)-induced Ca^2+^ signaling in TBC. Ca^2+^ imaging studies were performed in calcium-containing buffer. The changes in free intracellular Ca^2+^ concentrations (Δ*F_340_*/*F_380_*) were monitored under the Nikon microscope (TiU) by using S-fluor 40× oil immersion objectives. Graphs show the increase in [Ca^2+^]_i_ in taste bud cells freshly isolated from WT mice following addition of anandamide (AEA, 5 µM) with or without preincubation (20 min) with DB-cAMP (1 mM) or preincubation (20 min) with U73122 (10 µM) (left panel). The arrowhead indicates when the test molecules were added. Changes as histograms (right panel) in Δ*F_340_*/*F_380_* Ratio induced by anandamide (AEA, 5 µM) in WT mice TBC after a preincubation with or without DB-cAMP (1 mM) or U73122 (10 µM). Values are expressed as mean ± SEM (*n* = 6).

Finally, in order to validate our model, we investigated the downstream CB_1_R pathway elicited by AEA treatment. Here, we used DB-cAMP, a cAMP analog and a phosphodiesterase inhibitor, and U73122, a phospholipase C inhibitor. We observed that DB-cAMP did not significantly alter the Ca^2+^ response induced by AEA. Conversely, a pretreatment with U73122 significantly decreased the Ca^2+^ response, indicating that the Ca^2+^ response triggers a PLC-dependent Ca^2+^ signaling (Figure 7).

## 4. Discussion

Williams and Kirkham [19] demonstrated that CB_1_R is responsible for increased food intake, induced by an endocannabinoid agonist. Later on, Yoshida et al. [7] revealed that endocannabinoids enhanced the gustatory responses to sweet tastants via CB_1_R. Indeed, the activation of endocannabinoid system (ECS) appears to be associated with hyperphagia and a preference for palatable food. Interestingly, CB_1_R are also expressed in a subset of taste bud cells [7]. We report here that CB_1_R^−/−^ mice displayed no preference for fat solutions compared to WT mice. The same behavior was also observed when WT mice were treated with rimonabant, a CB_1_R blocker, confirming the role for this receptor in the detection of dietary lipids. We have employed LA as a candidate for LCFA because this fatty acid is abundantly present in Western food; however, it is possible that the saturated fatty acids like palmitic acid (PA) might also initiate the same gustatory response. Indeed, we have shown previously that LA and PA triggered the same increases in [Ca^2+^]_i_ in mouse taste bud cells [6]. 

In the present study, for behavioral experiments, we used whole body knockout mice for CB_1_R, and it is possible that the hypothalamic cannabinoid system, via the dopaminergic area, might be involved in fat taste preference [28]. Nonetheless, we sought to elucidate cellular mechanisms in the modulation of fat preference. We first tested the hypothesis whether there is an alteration in CD36 and GPR120 protein in TBC of CB_1_R^−/−^ mice. In our study, CD36 and GPR120 protein expressions were not altered by the absence of CB_1_R, suggesting that the absence of preference for fatty solutions may be due to altered downstream signaling. Moreover, we checked the delivery of linoleic acid under both conditions, and we observed identical uptake of exogenous fatty acid.

Previous studies indicated that both CD36 and GPR120 activation by a LCFA triggered mobilization of [Ca^2+^]_i_ from the intracellular endoplasmic reticulum Ca^2+^ pool during fat taste perception [9,29]. In our study, we show, for the first time, that LA-mediated increase in [Ca^2+^]_i_ was altered when CB_1_R was inactivated by rimonabant or by the absence of CB_1_R. In addition, the CB_1_R agonist ACEA also increased calcium flux per se in TBC, albeit with lower potency than LA. However, the effect of ACEA was maintained in TBC from CB_1_R^−/−^ mice, raising the possibility that the increase in [Ca^2+^]_i_ could be mediated by the receptors other than CB_1_R, for example, TRPV1. Indeed, it has been shown that activation of TRPV1 by endocannabinoids induces calcium signaling [30,31]. Besides, blockade of TRPV1 with A784168 totally abolished [Ca^2+^]_i_ response induced by ACEA, indicating that the residual calcium signal observed in CB_1_R^−/−^ TBC with ACEA may be due to TRPV1 activity. Furthermore, it appears that the CB_1_R-coupled downstream signaling is PLC-dependent, in accordance with the observations of De Petrocellis et al. [32]. However, it remains to be elucidated in future whether anadamide, employed in the present study, activates the Gβγ subunit of CB_1_R, and activates PLC via PI-3-kinase pathway. As a whole, our data indicate that CB_1_R may play a crucial role in fat taste perception by modulating calcium signaling.

As previously described, GLP-1^−/−^ mice have reduced taste responses to dietary fat, suggesting that orosensory detection of LCFA could be associated to the secretion of lingual GLP-1 [13]. Data reported herein showed that the secretion of active GLP-1 induced by LA is strongly decreased in CB_1_R^−/−^ mice suggesting the existence of a link between CB_1_R signaling and GLP-1 production. Hence, CB_1_R activation may stimulate proglucagon and GLP-1r production and, therefore, modulate perception threshold of LCFA. Further investigations are needed to explore the possibility whether GLP-1 secretion is stimulated via [Ca^2+^]_i_ signaling in TBC or by other mechanisms [33].

In conclusion, the present report shows that CB_1_R influences fat taste perception via regulating calcium signaling in TBC. It is proposed that CB_1_R activation induces a [Ca^2+^]_i_ response that strengthens fat perception, that is mediated by CD36. Activation of ECS could, thereby, increase sensory stimuli relaying palatability of foods and, ultimately, stimulate food intake. The physiopathological relevance of such a regulatory pathway is supported by the fact that ECS tone is increased in obesity. Hence, the ECS seems to emerge as a key modulator of oral sweet and fat detection and may represent a potential target for developing new anti-obesity strategies or, conversely, for enhancing food intake in the case of loss of appetite as it occurs in cachexia.
